# Surgical management of auricular keloids using the fillet flap technique

**DOI:** 10.1016/j.jdcr.2026.06.045

**Published:** 2026-06-27

**Authors:** Emily Deehan, Allison Eaton, Joseph Gofman, Daniel Fischer

**Affiliations:** a Department of Medical Education, Nova Southeastern University Dr. Kiran C. Patel College of Osteopathic Medicine, Fort Lauderdale, Florida; b Department of Dermatology, St. Barnabas Hospital, Bronx, New York; c Department of Dermatology, NYU Langone, New York, New York

**Keywords:** auricular keloid, earlobe keloid, fillet flap excision, keloid

## Background

We report a case that expands the limited literature on auricular keloid management, demonstrating effective control of a large, treatment-resistant lesion with fillet flap excision combined with intralesional corticosteroid adjuvant therapy. The fillet flap technique, first described for earlobe keloids in 2004, involves excision of the keloid core (“core fillet flap” used interchangeably with “fillet flap in practice and literature) while preserving a thin, viable skin flap of epidermis and dermis. This preserved flap allows for tension-free primary closure using native tissue and can be combined with adjuvant therapies such as corticosteroid injections and compression clamps to minimize recurrence. The approach is particularly valuable for auricular keloids, which comprise nearly one-quarter of all keloid cases and frequently arise following ear piercing.^1^ Auricular lesions pose a distinct challenge due to their cosmetic significance, high visibility, and propensity for recurrence.

Keloids are benign fibroproliferative scars marked by excessive collagen deposition and extension past the original wound margins, lacking spontaneous regression. This reflects aberrant transforming growth factor-β and immune cell-fibroblast crosstalk that drive persistent fibroblast activation and extracellular matrix accumulation.^1^ Genetic and immunologic contributions have also been identified, underscoring the heterogeneity of keloid disease across populations, specifically in the black population.^1^

## Case report

**Fig 1:**
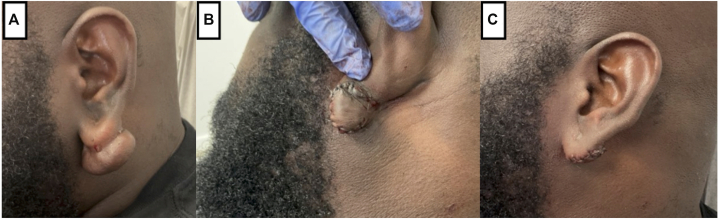
**A-C,** Presurgical presentation of earlobe keloid **(A)**. Postsurgical anterior view of the earlobe following fillet flap excision **(B)**. Postsurgical anterior view of the earlobe following fillet flap excision **(C)**.

We present the case of a 26-year-old, Fitzpatrick skin type V, male with a 4 × 2 cm keloid on the left earlobe, which had been present for two years (Fig 1, *A*). He had previously received multiple intralesional triamcinolone injections, consisting of 2 mL of 40 mg/mL administered every 6-8 weeks over an 18-month period, with minimal clinical improvement in size or induration. After preoperative evaluation of the lesion’s size, location, and morphology, photographic documentation, and informed consent, the patient underwent fillet flap excision under local anesthesia. The overlying skin was incised and carefully dissected to preserve a thin, viable posterior flap, while the underlying fibrocollagenous scar tissue was excised in its entirety, taking care not to extend into normal tissue. Redundant skin was trimmed as needed, and the preserved flap was re-approximated with fine interrupted sutures (5-0 nylon) to achieve a tension-free closure and maintain the earlobe contour (Fig 1, *B* and *C*). An intraoperative intralesional corticosteroid (triamcinolone 10 mg) was administered to the wound margins. Postoperatively, a firm, evenly distributed pressure dressing was applied, and the patient continued intralesional triamcinolone 10 mg every 2 weeks for 2 months. The patient initiated use of a metal spring-pressure (clip-on) earring approximately 2 weeks postexcision, wearing it for about 12 hours, 5-7 days weekly for 3 months to provide sustained compression.

At the two-month in-office follow-up, no evidence of keloid recurrence was observed. At the six-month in-office follow-up, examination continued to demonstrate no recurrence with minimal residual scarring, and the patient reported improved quality of life. While longer-term surveillance is warranted given the natural history of keloids, these findings provide objective follow-up beyond the early postoperative period. This multimodal approach, combining precise surgical technique, corticosteroid therapy, and pressure therapy, achieved a favorable cosmetic outcome during the reported follow-up period.

## Discussion

The management of auricular keloids remains a persistent clinical challenge due to their high recurrence rate and cosmetic impact. Although first-line therapy for keloid removal focuses on intralesional corticosteroid injection, results are variable and often unsatisfactory, prompting the necessitation of surgical intervention.^2^

Surgical excision with a fillet flap offers advantages, including a low recurrence rate of approximately 9.5% when combined with intralesional steroids, with some reports noting rates approaching 0% when combined with intralesional steroids and pressure therapy.^3^^,^^4^ Recurrence does not appear to be significantly affected by patient age, sex, treatment history, or lesion size and ear location.^5^ Reported risks include wound dehiscence, depigmentation, flap necrosis, and intralesional abscess formation.^5^

Other therapies include radiation-based protocols and shave removal with contouring. Radiation monotherapy reduces keloid thickness but is associated with a 37% recurrence rate.^6^ When combined with surgical excision, adjuvant radiation therapy yields an overall recurrence rate of 5.6% at a median follow-up of 40 months, with outcomes varying by dose.^7^ Shave removal with contouring focuses on debulking the keloid while leaving the core intact. Although it is a faster outpatient procedure, recurrence remains high due to persistence of the underlying core.^4^ Evidence for the use of pressure devices as monotherapy for keloids is limited, as they are primarily used as post-treatment adjunctive therapy, with late-intervention pressure garment therapy showing only a 4.5% reduction in hypertrophic scar severity (Vancouver Scar Severity scale).^8^

Immunomodulators commonly used as adjuvant therapies include imiquimod, systemic pentoxifylline, verapamil, and intralesional steroids. Imiquimod, a toll-like receptor 7 agonist, is convenient due to topical application but is not routinely recommended because of prolonged treatment duration, a relatively high rate of hyperpigmentation, and limited published evidence. Pentoxifylline, a nonselective phosphodiesterase inhibitor, remains largely investigational with limited data from small, single-center studies. Intralesional verapamil has been better investigated, but overall appears less effective than intralesional steroids. Two recent studies have shown intralesional verapamil is less effective than intralesional steroids, with 0% keloid clearance in comparison to 75% for triamcinolone (*P* < .01) and poorer improvements in scar pliability and vascularity (*P* < .05).^9^^,^^10^ The authors note that current evidence remains insufficient and further research into verapamil is needed.^10^

## Conclusion

Multiple treatment options exist for auricular keloids, but no single approach has a definitive superiority. Although the fillet flap technique is an established surgical option, it remains underutilized in dermatologic surgery. This case supports its feasibility and favorable short-term outcome; however, our conclusions are limited by single-patient design and short-term follow-up. While existing literature suggests that fillet flap excision with adjuvant therapy may reduce auricular keloid recurrence, it remains limited by a lack of long-term follow-up data across studies, which restrict definitive conclusions regarding durability of outcomes. Future research should focus on large-scale, randomized trials to validate these outcomes and establish standardized protocols. Comparative trials evaluating different adjuvant therapies in conjunction with surgical techniques are also needed to determine the most effective combinations with minimal risk.

## Conflicts of interest

None disclosed.
